# Meta-Analysis of Microarray Studies Reveals a Novel Hematopoietic Progenitor Cell Signature and Demonstrates Feasibility of Inter-Platform Data Integration

**DOI:** 10.1371/journal.pone.0002965

**Published:** 2008-08-13

**Authors:** Davendra Sohal, Andrew Yeatts, Kenny Ye, Andrea Pellagatti, Li Zhou, Perry Pahanish, Yongkai Mo, Tushar Bhagat, John Mariadason, Jacqueline Boultwood, Ari Melnick, John Greally, Amit Verma

**Affiliations:** 1 Albert Einstein College of Medicine, Bronx, New York, United States of America; 2 John Radcliffe Hospital, Oxford, United Kingdom; University of Manchester, United Kingdom

## Abstract

Microarray-based studies of global gene expression (GE) have resulted in a large amount of data that can be mined for further insights into disease and physiology. Meta-analysis of these data is hampered by technical limitations due to many different platforms, gene annotations and probes used in different studies. We tested the feasibility of conducting a meta-analysis of GE studies to determine a transcriptional signature of hematopoietic progenitor and stem cells. Data from studies that used normal bone marrow-derived hematopoietic progenitors was integrated using both RefSeq and UniGene identifiers. We observed that in spite of variability introduced by experimental conditions and different microarray platforms, our meta-analytical approach can distinguish biologically distinct normal tissues by clustering them based on their cell of origin. When studied in terms of disease states, GE studies of leukemias and myelodysplasia progenitors tend to cluster with normal progenitors and remain distinct from other normal tissues, further validating the discriminatory power of this meta-analysis. Furthermore, analysis of 57 normal hematopoietic stem and progenitor cell GE samples was used to determine a gene expression signature characteristic of these cells. Genes that were most uniformly expressed in progenitors and at the same time differentially expressed when compared to other normal tissues were found to be involved in important biological processes such as cell cycle regulation and hematopoiesis. Validation studies using a different microarray platform demonstrated the enrichment of several genes such as SMARCE, Septin 6 and others not previously implicated in hematopoiesis. Most interestingly, alpha-integrin, the only common stemness gene discovered in a recent comparative murine analysis (Science 302(5644):393) was also enriched in our dataset, demonstrating the usefulness of this analytical approach.

## Introduction

Microarray-based studies of global gene expression have led to dramatic advances in our understanding of various biological processes. This technology has become one of the most rapidly growing investigational methods in medical research and numerous studies have been completed using this method. There are many available platforms [1] for microarray analysis, and newer technologies and better gene annotations have led to constant refinement of these platforms. This has resulted in a large amount of data in public repositories, like the Gene Expression Omnibus [2]. Meta-analysis of these data has the potential to yield important biological information, but is hampered by technical issues. Cross-platform comparability has been a major hindrance to this approach. This problem arises because matching probe-sets across platforms is a difficult task. Different platforms use different probe lengths and sequences, and mapping them to one common gene or set of genes is beset with problems. Another limitation is different gene annotations used by different platforms. The nucleic acid sequences for various species are submitted to and maintained in the GenBank® database by the National Center of Biotechnology Information (NCBI) [3]. There are different annotation methods in use to parse these sequences into genes or gene clusters. UniGene is one method for partitioning GenBank nucleic acid sequences into unique gene-oriented clusters, each of which represents a unique gene. These UniGene identifiers (IDs) are created by finding transcript sequences that match distinct transcription areas or genes. UniGene IDs have been used as the matching criterion to merge data across various platforms, but this has led to a substantial portion of the data remaining unmatched in previous studies [4], [5], [6], [7], [8]. Recent approaches have tried using Reference Sequence (RefSeq) IDs as the matching criterion [9]. RefSeq is a public access database, also maintained by NCBI. This database is built by using sequence data from GenBank, EMBL Data Library (UK) and DNA Data Bank (Japan) [10]. This set is also constantly updated, and input from various investigators is also used to maintain this set. Since both UniGene and RefSeq are billed as non-redundant sets of transcript IDs, and have been used in prior studies with mixed results, it is still unclear as to which approach is better.

We attempted to conduct a meta-analysis of all gene expression studies using hematopoietic progenitor cells to determine a gene expression signature characteristic of these cells. Our aim was to integrate data from all studies that used normal hematopoietic progenitors and stem cells into a unified normalized database. This was done using both UniGene as well as RefSeq gene IDs to assess which identifier provides the best yield. Our results show that experimental conditions, laboratory where the experiments were performed and different microarray platforms can result in significant variability in gene expression patterns from similar sources of cells. In spite of experimental variability, meta-analytical studies do have the power to discriminate biologically distinct tissues on the basis of their normalized gene expression patterns. Gene expression datasets from similar cells of origin cluster together despite diseased phenotypes and genetic alterations. The similarity seen among gene expression profiles of leukemias, myelodysplasia and normal hematopoietic progenitors, when compared to non-hematopoietic tissues, validates the functional discriminatory power of this meta-analysis. Finally, analysis of merged normal hematopoietic progenitor cell gene expression datasets led to the discovery of a common gene expression signature characteristic of these cells. Genes that are most uniformly expressed in normal hematopoietic tissues and at the same time being differentially expressed as compared to other normal tissues were found to be involved in important biological processes such as hematopoiesis and development. The expression patterns of these genes were validated in a different microarray platform using material from three different hematopoietic progenitor and stem cell experiments.

## Methods

### Data collection

**Figure 1:**
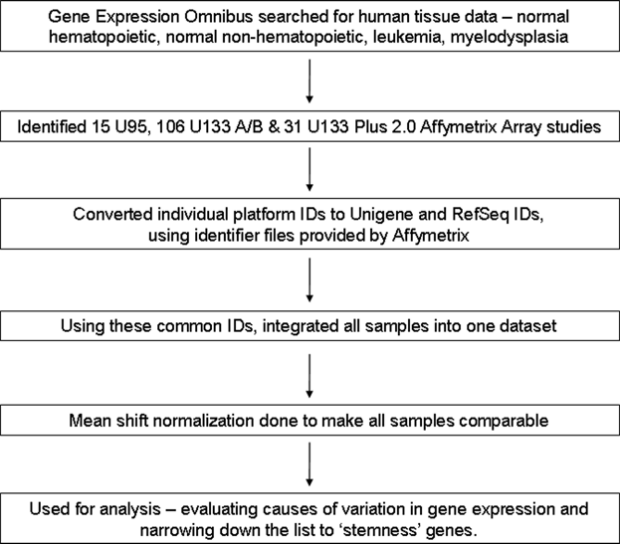
Schema of data collection and analysis.

Normal hematopoietic cell gene expression data were collected from the NCBI's Gene Expression Omnibus (GEO) database (Figure 1). Bone marrow, hematopoietic, CD34 and stem cells were used as search terms to locate datasets containing gene expression profiles of normal human hematopoietic cells. Normal bone marrow/peripheral blood CD34 profiles used as controls in studies of leukemia and other hematological diseases were also included. Most studies used the Affymetrix U95, the U133A/B and the U133 Plus 2.0 Array Platforms. A handful of studies using older Affymetrix platforms, like the HG-Focus Target Array and the Full Length HuG Array, were discarded because combining data from these yielded a lot of non-matching probe-sets.

Gene expression data for other normal tissues assayed on the same platforms were also obtained from GEO. In a study where multiple sets were available, we picked one set each for every tissue, again to minimize correlation within individual datasets. These were picked using computer-generated random numbers.

To obtain diseased stem cell data, we identified a few studies with multiple datasets on myelodysplasia, acute myeloid leukemia (AML) and acute lymphoblastic leukemia (ALL) samples. Where several datasets were available per study, we picked a subset, again using random numbers, to obtain about 10 samples per each study. Table 1 shows the details of these datasets [11], [12], [13], [14], [15], [16], [17], [18], [19].

**Table 1:** Sources of data for the meta-analysis*

Author	Source of cells	No. of datasets	Platform
Sternberg A, et al [11]	CD34, MDS	22	U133 A/B
Oswald J, et al [12]	CD34	3	U133 A/B
Su AI, et al [13]	CD34, various normal NHTs	19	U133 A/B
Eckfeldt CE, et al [14]	CD34	18	U133 A/B
Bhatia M, et al (GEO)	CD34	15	U133 A/B
Pellagatti A, et al [15]	CD34, MDS	22	U133 Plus 2.0
Breit S, et al [16]	Bone marrow	9	U95
Ge X, et al [17]	Various normal NHTs	19	U133 A/B
Gutierrez NC, et al [18]	Bone marrow (AML)	9	U133 A/B
Roth RB, et al (GEO)	Various normal NHTs	7	U133 Plus 2.0
Cheok MH, et al [19]	Bone marrow (ALL)	6	U95

* NHTs: Non-hematopoietic tissues, GEO: Gene Expression Omnibus database set,

MDS: Myelodysplastic syndrome, AML: Acute myeloid leukemia, ALL: Acute lymphoblastic leukemia

Numbers in brackets are reference numbers.

### Integration of datasets

Initially, we used the comparison spreadsheets provided by the chip manufacturer, Affymetrix [20]. These files link the probe-set IDs of various platforms. However, the yield therein was poor. For example, using the link file between the U133 and the U133 Plus platforms, 44635 U133 IDs matched to only 9908 U133 Plus IDs. Therefore, UniGene and RefSeq IDs were evaluated as variables to cross link data from various platforms. Individual probe-set IDs for each platform were linked to the corresponding UniGene IDs using annotation files, again provided by Affymetrix [20]. These UniGene IDs were then used to combine data across the three platforms. Once probe-set IDs and their expression values were combined, the expression value for each UniGene ID was obtained. In many instances, more than one probe-set matched to the same UniGene ID, resulting in multiple expression values for each such ID. In such cases, the expression value was calculated as the mean of the various values for each UniGene ID.

Probe-set IDs which did not match to any UniGene ID were dropped. Also, if any UniGene ID had data for only one platform, it was dropped, as it was considered to not match across at least two of the three platforms. Moreover, in many cases, one probe-set ID matched to more than one UniGene ID. In such cases, each UniGene ID was considered to have the same gene expression value and the data were expanded accordingly.

An identical process was used to merge data across platforms using RefSeq ID as the match identifier, instead of UniGene ID. Of interest, RefSeq IDs can be either protein IDs or transcript IDs. Protein IDs provided slightly better results (as detailed further) than transcript IDs, and were therefore used in this study.

### Data analysis

Once expression values for each UniGene or RefSeq ID were obtained, these were used to do the analysis. First, the datasets were normalized using quantile normalization to ensure that inherent large-scale expression differences in the datasets based on different sources and laboratories were minimized. Unsupervised hierarchical clustering using average linkage with (1 - Pearson correlation coefficient) as the distance measure was done for each of the three ‘types’ of tissue – normal hematopoietic cells, normal non-hematopoietic tissues and diseased hematopoietic cells. This allowed us to look at how the datasets cluster – whether by platform, laboratory, experiment or otherwise.

To determine a gene signature for hematopoietic progenitor and stem cells, we used the datasets derived from 57 CD34+ sets, as whole bone marrow sets may not be a true reflection of these progenitors, being as they are a mixture of various cell types. To find out which genes were most consistently expressed across these samples, we used the coefficient of variation – defined as the standard deviation divided by the mean – of the expression values for each ID, calculated across all stem cell samples. The coefficient of variation was used to incorporate consistency in gene expression as well as “enrichment” of genes in the hematopoietic progenitor cells. Prior studies have used similar reasoning [21].

We then used this set of consistently expressed genes and compared their expression in normal hematopoietic progenitors versus that in non-hematopoietic tissues, to identify which genes could differentiate these two tissue sources. This was done using significance analysis of microarrays (SAM) [22], [23]. Similarly, normal hematopoietic progenitor gene expression was compared to diseased hematopoietic data, to identify a subset of genes that may be most relevant to hematological stem cell disorders. All IDs with missing values for any of the samples were deleted.

All data analyses were done using SAS (SAS Institute, Cary, NC), the R language and ArrayAssist Expression software package (Stratagene Corporation, La Jolla, CA).

## Results

### Integration of data using protein identifiers

A total of 66 individual normal hematopoietic cell expression profiles were identified in NCBI's GEO database (Table 1). Nine were derived from whole bone marrow samples and 57 were from selected CD34-positive cells. These studies were performed on 3 different microarray platforms (Table 2). Since the probe-set identifiers and complementary oligos were different on these platforms, we integrated the data using both UniGene and RefSeq protein IDs (Figure 1, showing schema).

**Table 2:** Platform and tissue type for various datasets.

	U95	U133 A/B	U133 Plus 2.0	Total
Normal hematopoietic stem cells	9	46	11	66
Normal tissues, non-hematopoietic	0	36	7	43
Diseased hematopoietic stem cells	6	23	11	40
Total	15	105	29	149

The Affymetrix annotation files yielded 12,626 unique probe-sets in the U95 platform, 44,761 in the U133 A/B platform and 54,676 unique probe-sets in the U133 Plus 2.0 platform. Using UniGene IDs as the matching criterion, 11,635, 40,787 and 45,867 probe-sets matched to at least one other platform, respectively. After combining data from all the three platforms, we ended up with a total of 20,717 UniGene IDs. Since one probe-set can match to more than one UniGene ID and vice versa, a relatively small number of U95 probe-sets matched to 20,717 UniGene IDs. Using RefSeq protein IDs as the matching parameter, 11,722, 37,395 and 42,462 probe-sets matched to at least one other platform, respectively. As many of the probe-sets from the two newer platforms were coding for the same protein, we ended up with a total of 28,497 unique RefSeq protein IDs that were common to all three platforms. After removing the probe-sets where expression values were missing for any dataset, a total of 8,598 unique UniGene IDs and 8,345 unique RefSeq IDs were obtained that were common to all platforms. These were quantile-normalized using ArrayAssist (Strategene Corporation, California, USA) to adjust for hybridization intensities and used for the meta-analysis.

### Experimental conditions, microarray platforms and source of cells can influence gene expression patterns

**Figure 2:**
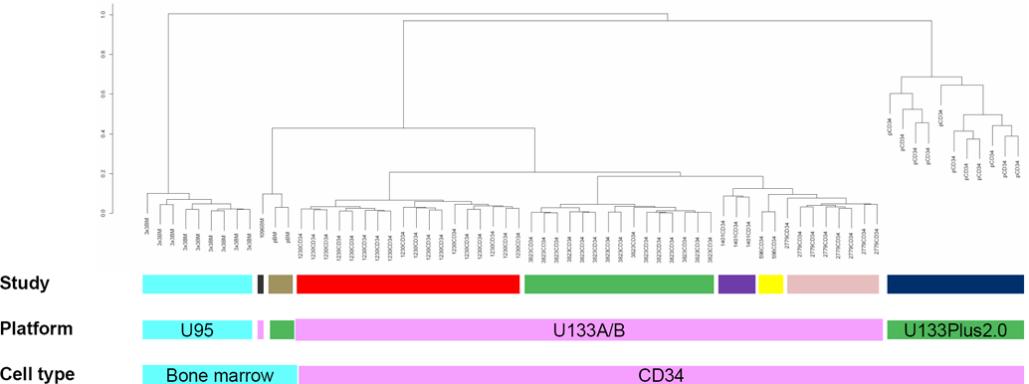
Normal bone marrow HSC clustering. Experimental conditions, microarray platforms and sources of cells influence gene expression patterns of normal bone marrow derived HSCs. Dendrogram of normal bone marrow derived hematopoietic cells based on unsupervised hierarchical clustering, using (1 - Pearson correlation coefficient) as the distance measure. Same color in each horizontal row indicates same group.

Sixty-six hematopoietic gene expression profiles from either whole bone marrow or selected CD34 cells were grouped using unsupervised clustering based on Pearson correlation coefficient. In spite of similar cell types, the studies grouped primarily based on the laboratory where the data was obtained from. The next level of clustering was defined by the microarray platform used for the studies. Barring two bone marrow samples from the Plus 2.0 platform that were similar to one bone marrow sample from the 133A/B platform, all the samples clustered depending on which platform they were from. The samples from the U95 platform stayed as a separate group (Figure 2). The last level of similarity was based on the exact source of the cells used for the RNA.

The correlation coefficients between various datasets validated the clustering order of laboratory, platform and source (Table 3). The correlation was strongest between samples obtained from the same laboratory/study, with a mean (median) absolute correlation coefficient of 0.87 (0.95). When the platform was the same, a slightly lesser though still strong correlation of 0.83 was obtained. These results illustrate that the cause of variability in gene expression studies can be due to experimental conditions/protocols used in individual laboratories, platforms used as well as sources of cells in that order.

**Table 3:** Pairwise absolute correlation coefficients for normal hematopoietic cell samples

	Mean (Range)	Median
Same study	0.87 (0.26–1.00)	0.95
Different study	0.35 (0.00–0.93)	0.04
Same platform	0.83 (0.26–1.00)	0.82
Different platform	0.02 (0.00–0.06)	0.01
Same cells (CD34 or BM)	0.58 (0.01–1.00)	0.79
Different cells	0.01 (0.00–0.01)	0.01

### Gene expression studies from biologically distinct tissue types can be compared despite varying platforms and experimental conditions

**Figure 3:**
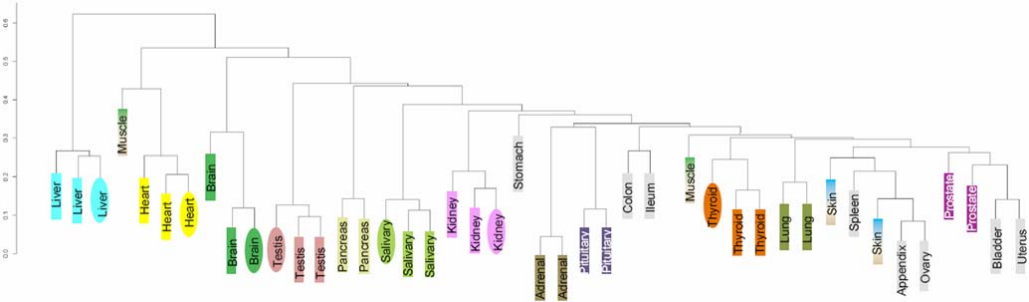
Distinguishing normal non-hematopoietic tissues. Despite differing platforms and experimental conditions, GE profiles can separate out normal tissues based on cell/tissue of origin. Dendrogram based on unsupervised hierarchical clustering, using (1 - Pearson correlation coefficient) as the distance measure. Rectangles indicate samples from the U133 A/B platform and ovals from the U133 Plus 2.0 platform. Triplicate sets of samples from human liver, heart, testis, kidney, etc. are from different studies, and their grouping together is a strong indicator of comparability across studies and platforms.

We next wanted to determine the degree of dissimilarity of hematopoietic datasets to gene expression (GE) datasets obtained from other biologically distinct tissues. GE profiles from human adrenal, appendix, brain, breast, colon, heart, kidney, liver, lung, ovary, pancreas, pituitary, prostate, salivary gland, skin, small intestine, smooth muscle, spleen, stomach, testis, thyroid, urinary bladder and uterus samples were obtained from the GEO database and used for this analysis. Unsupervised clustering showed that samples from the same tissue of origin clustered tightly together in spite of different platforms/laboratories used for the analysis (Figure 3). Clustering of triplicate sets of liver, heart, brain, salivary gland, testis, kidney and thyroid tissues from different laboratories and platforms clearly indicates that our analysis can detect the similarity of expression at the source tissue level. The mean (and median) correlation coefficients were also not very dependent on the laboratory/study or the platform (Table 4). The highest correlation was observed between similar tissues. These results demonstrate that despite inter-platform and inter-study variability, meta-analysis of gene expression profiles has the potential of revealing differences between tissues with a high degree of dissimilarity (Table 4).

**Table 4:** Pairwise absolute correlation coefficients for normal non-hematopoietic cell samples.

	Mean (Range)	Median
Same study	0.58 (0.27–0.91)	0.59
Different study	0.55 (0.23–0.95)	0.55
Same platform	0.58 (0.24–0.95)	0.59
Different platform	0.51 (0.23–0.88)	0.49
Same tissue	0.77 (0.68–0.95)	0.80
Different tissue	0.57 (0.23–0.88)	0.59

### Gene expression datasets from similar cells of origin can cluster together despite diseased phenotypes and genetic alterations

**Figure 4:**
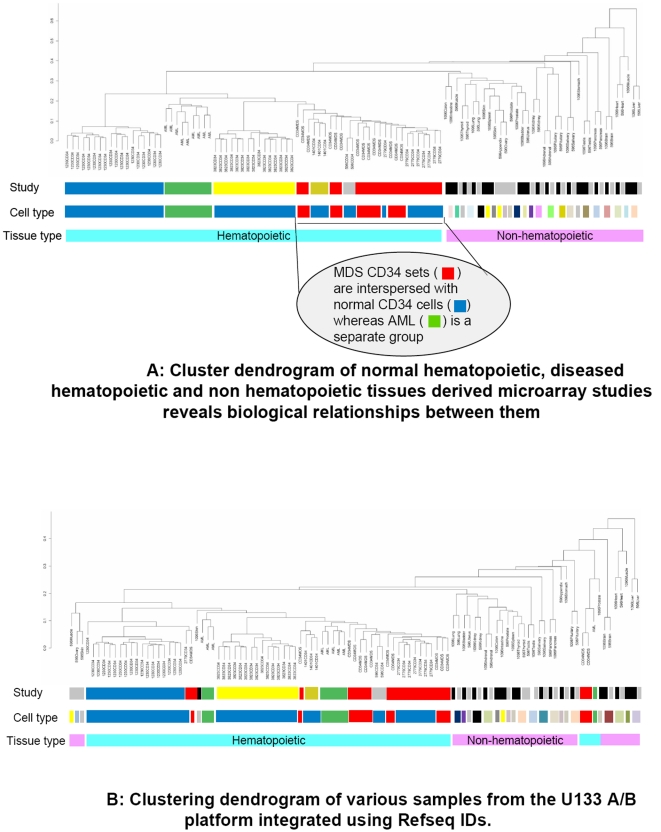
A: Biological relationships identified. Dendrogram of normal hematopoietic, diseased hematopoietic and non hematopoietic tissues GE profiles reveals biological relationships between them. MDS sets intersperse with normal hematopoietic tissues whereas AML samples are a separate group, exactly as their biological dissimilarity patterns. Dendrogram based on unsupervised hierarchical clustering, using (1 - Pearson correlation coefficient) as the distance measure. Same color in each horizontal row indicates same group. UniGene IDs were used for integrating data. B: Clustering using RefSeq IDs. Same clustering as in 4A, showing poorer performance of RefSeq IDs, compared to UniGene IDs, in uncovering biological relationships. Dendrogram based on unsupervised hierarchical clustering, using (1 - Pearson correlation coefficient) as the distance measure. Same color in each horizontal row indicates same group.

To further test the discriminatory ability of the meta-analysis, we next grouped datasets from hematologic malignancies with the normal hematopoietic and non-hematopoietic tissues analyzed within the same microarray platform (U133 A/B). We wanted to determine whether biological variability seen in hematopoietic stem cell disorders such as acute leukemias and myelodysplastic syndromes would be distinguishable in our analysis. Unsupervised clustering showed that even though diseased hematopoietic cells were separated from the normal cells, they were significantly more dissimilar to non-hematopoietic tissues (Figure 4A). In fact, some individual GE profiles from bone marrow CD34+ samples from myelodysplastic syndromes were very similar to normal CD34+ cells and clustered within their groups. We believe that this was a strong validation of our analytical approach as myelodysplasia is a preleukemic disorder with varying levels of pathology and can have cases that are genetically very similar to normal hematopoietic stem cells [24]. We did a similar analysis using RefSeq IDs as the matching criterion between different datasets. Interestingly, clustering using RefSeq IDs provided more heterogeneous results (Figure 4B) and grouped non-hematopoietic tissues along with hematopoietic tissues, thus demonstrating that UniGene IDs are better at discriminating biological subsets.

### Hematopoietic progenitor and stem cell signature

After validating the strength of the meta-analysis, we wanted to determine a gene expression signature of hematopoietic progenitors. Using the lowest 20^th^ percentile, to obtain the best possible initial yield, a total of 1,719 genes were obtained with a low coefficient of variation among the 57 CD34+ GE profiles (range 0.15–0.39). These were the genes deemed to be most characteristic of the stem and progenitor cells as their expression was most consistently enriched among all the samples.

**Figure 5:**
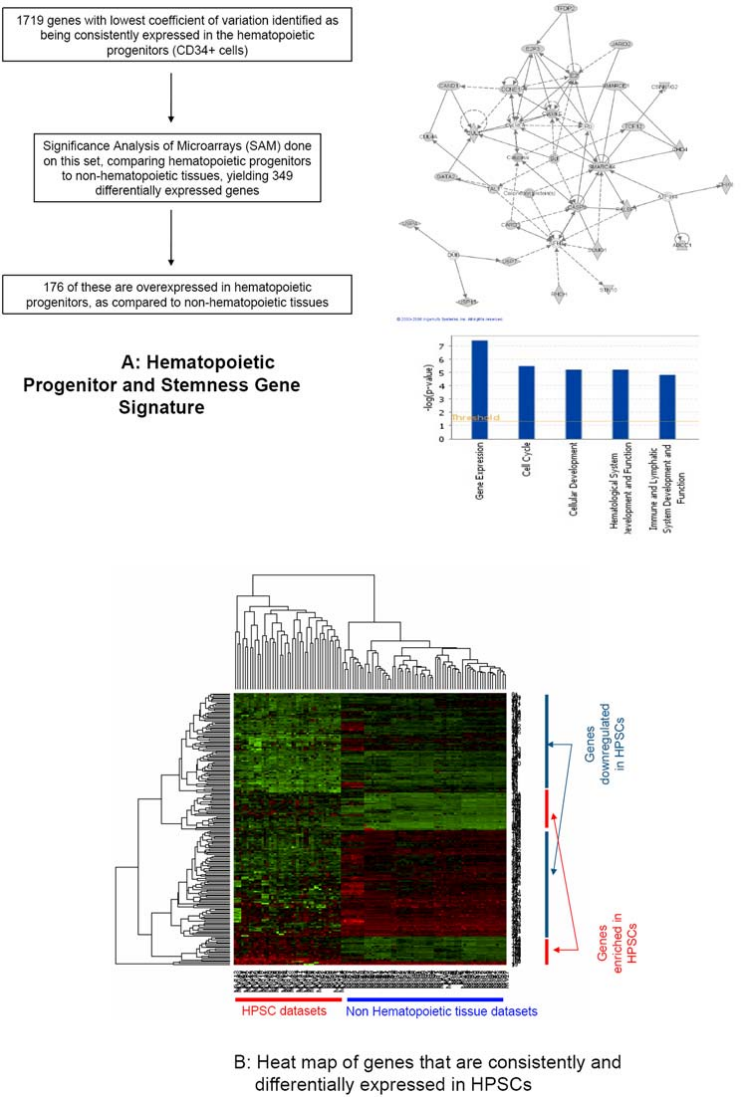
A: “Stemness genes”. 349 UniGene IDs were identified as being consistently expressed amongst the normal hematopoietic cells and differentially expressed between hematopoietic and non-hematopoietic cells. Genes enriched in hematopoietic progenitor and stem cell datasets were involved in important functional pathways in the cell, including drug metabolism, hematological system development, cell signaling and cancer and cell death, as shown in the bar graph alongside. One such network is shown, which includes the GATA2, Cyclin E and SMARCE1 genes. B: Heatmap of “stemness” genes. 349 Unigene IDs were identified as being consistently expressed amongst the normal hematopoietic cells and differentially expressed between hematopoietic and non-hematopoietic cells. Out of these, 176 genes were enriched in HSC datasets when compared to other tissue types.

Using this list of genes, we next determined the genes that were able to discriminate normal hematopoietic and non-hematopoietic cells by using significance analysis of microarrays (SAM). We used 100 permutations to compute the expected significance ‘score’, and a false discovery rate (FDR) of 0.29% was achieved by using the lower- and upper-most 10% of genes. A total of 349 genes were called as significant (Figure 5).

To better understand how differentially expressed genes were integrated into specific regulatory and signaling pathway networks, we used Ingenuity Pathway Analysis (Ingenuity Systems, Redwood City, USA). Functional analysis of overexpressed genes indicated that this list is highly enriched for proteins involved in hematopoiesis and cell cycle, further validating our approach (Figure 5, Table 5). Several of these genes have already been described to have important roles in development of the hematologic system. In addition, our analysis revealed a variety of novel functional genes like SWI/SNF family member 4, SMARCE1 and Septin 6. Many of the genes identified in our database were also found to be enriched in 3 independent HSC studies performed in our laboratory using a different Nimblegen platform (Supplementary Table S1). Cross validation suggests that these genes need to be tested as potential markers of HSCs and may have functionally important roles in hematopoiesis. We also found 171 genes to be differentially underexpressed in hematopoietic progenitors (Table 6). Our database and integration files will be online in a searchable format to aid other hematology and stem cell researchers (http://greallylab.aecom.yu.edu/).

**Table 5:** ‘Stemness genes’*

Major functions	Well-annotated genes
Gene Expression, Cell Cycle, Cellular Development,	ABCC1, CASP8, CSNK1G2, E2F3, GATA2, JARID2, RALBP1, SMARCA4, SMARCE1, STK10, SUMO1, TAL1, TCF12, TFDP2, USP4, USP7
Cell Morphology, Cellular Assembly and Organization, Cell Signaling	C1ORF2, GLIPR1, HSPA9, ING2, LPIN1, MAP3K4, MAP4K1, NCK1, NFATC1, PAK2, PPM1F, PPP3CA, TP53, UBE3A, ZNF84, BRPF1, EWSR1, HSPA4, LYN, MAPKAPK5, PHF21A, PTEN, TIMM17A, TROVE2
Cancer, Cellular Growth and Proliferation,Tumor Morphology	ATP6V0A2, CD47, HNRPUL1, MLLT10, MPHOSPH9, MTR, PDS5A, SEC63, SH3BGRL
Others	TIPRL, TSR1, TXNDC9, SFRS17A, CENTB2, THOC2, KIAA0368, PAX3, TFIP11, TUFT, FMR1, NUFIP1

* Some important genes differentially **over-expressed** in hematopoietic progenitors, as compared to non-hematopoietic tissues

**Table 6:** Genes quiescent in HSC progenitor cells*

Major functions	Well-annotated genes
Skeletal and Muscular System Development, Function and Disorders, Genetic Disorders	ADD1, APBB3, ARHGAP1, ATP2A2, BCL2L2, BGN, CALCOCO1, CALD1, CALM1, COL18A1, COL6A1, DDR1, ESRRA, FMOD, MYH9, NCOA1, PFN2, PXN, RHOC, SQSTM1, TPM1
Cellular Assembly and Organization, Cellular Function and Maintenance, Cell Signaling	APP, CADM1, CD59, CLSTN1, ERBB2, F8, FLOT1, GDI1, IKBKG, MAPK13, MYO1C, NDRG2, NFE2L1, PTRF, RAB5B, RAB5C, SFRP1, SHC1, SPTAN1, WFS1
Protein Degradation, Cellular Movement, Cell Morphology	ARF3, ARFIP2, CES2, COL1A1, CTNND1, EIF4G1, GSK3A, GSTA1, GSTM2, KIF5C, MFN2, MMP14, PAPSS2, PCDHGC3, PTPRF, SDC1, TIMP3, TSPAN3
Others	CDC42EP4, CHST10, DEFB1, FKBP1A, HDLBP, LPP, S100A13, TEGT, AKAP1, CLOCK, JAM3, PCTK1, TLE2, TMPRSS6, TNFAIP1, TRIP10, USP13, SPOCK2

* Some important genes differentially **under-expressed** in hematopoietic progenitors, as compared to non-hematopoietic tissues

## Discussion

Microarray analysis of global gene expression has led to rapid advances in our understanding of various physiological and pathological processes. Although many hundreds of studies have been done, doubts have been raised about the reproducibility and applicability of this data [25], [26], [27], [28]. Inter-study variability can be attributed to differing probes on the arrays, different protocols for RNA extraction, labeling and hybridization, and differences in the quality of cells. In spite of these factors, a number of studies have also demonstrated reproducibility of microarray studies performed at different platforms and laboratories, though most used the same source of RNA for these analyses [29], [30], [31]. The MicroArray Quality Control consortium (MAQC) was formed to address these questions and recently reported that reproducibility can be enhanced by better matching of microarray probes between platforms [32]. They concluded that matching probe-sets within the same exons and using similar experimental protocols can lead to more reproducible results when performed on major commercial microarray platforms. Our results take these findings a step further and demonstrate that GE studies done using different platforms and distinct sources of material have the power to discriminate between biologically distinct tissues and thus can also be used to analyze various scientific questions. Earlier attempts to address study specific biases have used statistical algorithms including ANOVA based correction models [33], [34]. We did not use these algorithms as we found adequate discrimination between biologically distinct tissues, demonstrating that the degree of differential gene expression is so large that it is found even in presence of possible study-specific biases. It is possible that some of the more subtle results seen in our analysis, however, may prove artificial once these biases have been removed by appropriate methods.

Furthermore, this meta-analysis can be accomplished simply by using UniGene and RefSeq identifiers as common variables between array platforms, though UniGene is shown to be slightly better at achieving this discrimination in our dataset. This difference between UniGene and RefSeq results, albeit small, is likely due to the different methods of identifying and assigning transcripts used in the process, and has been observed in prior studies also [4], [5], [6], [7], [8], [9], [10]. Even though we did observe variability due to different laboratory protocols as seen by previous studies, a superior correlation between tissues with similar sources of cells was able to surpass this limitation and make the meta-analysis scientifically useful.

Our study demonstrated that results obtained through this approach can be reconciled with the biology of hematopoietic cells and malignancies thereof. For example, samples from acute myeloid leukemia and myelodysplasia were found to be transcriptionally closer to normal hematopoietic cells than non-hematopoietic cells, even though these studies are done in many different laboratories. MDS is a preleukemic disorder of varying grades of pathology and can have an indolent course in most patients [15], [24]. The fact that MDS samples clustered with normal hematopoietic samples in some cases shows that our analysis can interpret biological relationships even between studies performed by different experimental protocols and laboratories.

After demonstrating that our approach can be used to biologically characterize sources of cells, we attempted to use this database to discover gene signatures characteristic of hematopoietic progenitor and stem cells. Due to the heterogeneity of our source dataset, we imposed very stringent criteria to discover genes characteristic of hematopoietic progenitors. Out of the 349 genes that were differentially expressed in normal progenitors, 124 are differentially expressed in diseased hematopoietic cells, demonstrating that hematologic malignancies result in disruption of important functional genes. Our search strategy yielded several genes that were consistently enriched in normal hematopoietic GE datasets and were found to be involved in cell cycle, growth, development and hematopoiesis by functional pathway analysis. Recent studies have supported similar comparative approaches for more accurate and valid gene target discovery [21], [26], [35]. Two recent seminal studies searched for gene signatures of stem cells by comparing genes enriched in hematopoietic, neural and embryonic stem cells and arrived at a total of 283 and 230 common ‘stemness’ genes respectively [21], [35]. Even though the experimental techniques and cell types in these two papers were similar, an initial comparative analysis showed that only 7 ‘stemness’ genes were common between these two studies. Comparison to a subsequent third analysis [26] showed even less overlap, with only one gene being consistently enriched between these three independent similar studies. Repeat analysis done using different statistical methods did lead to more gene overlap, but the final conclusion was that gene array studies of stem cells are influenced by cell purity and can be contaminated by a high level of non-specific observations in the data. Consequently, the authors determined that commonly expressed genes among different studies may be better representatives of functionally important stemness genes. Thus, meta-analytical approaches may be a way to separate functionally important information from experimental noise. As the genes discovered by our analysis are common in an extremely variable dataset, they may have a high chance of being characteristic of human HSCs. Most importantly, alpha-6 integrin, the one gene that was found be enriched in all three murine stem cell studies, is similar to alpha-4 integrin that was found to be enriched in our human dataset. Both of these integrins are known to be expressed on the surface of HSCs and are implicated in cell migration and homing to the bone marrow. The functional similarities between these two integrins and the concurrence of our findings with three landmark stemness gene studies published in the literature validate our analytical approach.

Our analysis also yielded a set of genes not previously implicated in hematopoiesis. Some of these genes have interesting functions and can be potential regulators of HSC function. SMARCE (SWI/SNF related, matrix associated, actin dependent regulator of chromatin, subfamily e/BAF57) is a key member of the mammalian SWI/SNF chromatin remodeling complex that is involved in transcriptional regulation [36]. SMARCE has been shown to mediate the interaction between the chromatin remodeling complex and transcription factors and thus could be partly responsible for the unique chromatin associated with stem cells [37]. Lyn kinase is a member of the src family of kinases and has been implicated in granulopoiesis and erythropoiesis and needs further exploration as a stem cell marker [38], [39]. Septin 6 is a member of a class of proteins involved in cell division, membrane trafficking and cytoskeletal organization. The roles of septins in hematopoietic stem cells remain unexplored [40]. Amyloid beta precursor protein is a cell surface protein with signal-transducing properties, and it is thought to play a role in the pathogenesis of Alzheimer's disease [41]. This protein can activate NEDD8, a ubiquitin-like protein required for cell cycle progression through the S/M checkpoint and thus can be potentially involved in cell cycle control of hematopoietic stem cells. The protein Dp-2 (E2F dimerization partner 2) belongs to a family of transcription factors that play an essential role in regulating cell cycle progression [42]. These transcription factors regulate the expression of numerous critical genes (e.g. cyclin E, CDC2, cyclin A, B-Myb, E2F1, and p107) involved in cell cycle progression as well as several enzymes (DNA polymerase α, thymidine kinase, and dihydrofolate reductase) required for DNA replication [42]. Thus Dp-2 could certainly be involved in stem cell regulation. In summary, our analytical approach provides a list of interesting genes for further scientific and functional validation. Additionally, this dataset can be used as an online resource for stem cell and hematology researchers as a control database for comparisons with disease state GE profiles done in their laboratories.

## Supporting Information

Table S1 (0.04 MB DOC)

## References

[1] Eisenstein M (2006). Microarrays: Quality control.. Nature.

[2] National Center for Biotechnology Information NLoM Gene Expression Omnibus. NCBI.

[3] Wheeler DL, Barrett T, Benson DA, Bryant SH, Canese K (2006). Database resources of the National Center for Biotechnology Information.. Nucleic Acids Res.

[4] Jarvinen AK, Hautaniemi S, Edgren H, Auvinen P, Saarela J (2004). Are data from different gene expression microarray platforms comparable?. Genomics.

[5] Kuo WP, Jenssen TK, Butte AJ, Ohno-Machado L, Kohane IS (2002). Analysis of matched mRNA measurements from two different microarray technologies.. Bioinformatics.

[6] Petersen D, Chandramouli GV, Geoghegan J, Hilburn J, Paarlberg J (2005). Three microarray platforms: an analysis of their concordance in profiling gene expression.. BMC Genomics.

[7] van Ruissen F, Ruijter JM, Schaaf GJ, Asgharnegad L, Zwijnenburg DA (2005). Evaluation of the similarity of gene expression data estimated with SAGE and Affymetrix GeneChips.. BMC Genomics.

[8] Warnat P, Eils R, Brors B (2005). Cross-platform analysis of cancer microarray data improves gene expression based classification of phenotypes.. BMC Bioinformatics.

[9] Ji Y, Coombes K, Zhang J, Wen S, Mitchell J (2006). RefSeq refinements of UniGene-based gene matching improve the correlation of expression measurements between two microarray platforms.. Appl Bioinformatics.

[10] Pruitt KD, Tatusova T, Maglott DR (2005). NCBI Reference Sequence (RefSeq): a curated non-redundant sequence database of genomes, transcripts and proteins.. Nucleic Acids Res.

[11] Sternberg A, Killick S, Littlewood T, Hatton C, Peniket A (2005). Evidence for reduced B-cell progenitors in early (low-risk) myelodysplastic syndrome.. Blood.

[12] Oswald J, Steudel C, Salchert K, Joergensen B, Thiede C (2006). Gene-expression profiling of CD34+ hematopoietic cells expanded in a collagen I matrix.. Stem Cells.

[13] Su AI, Wiltshire T, Batalov S, Lapp H, Ching KA (2004). A gene atlas of the mouse and human protein-encoding transcriptomes.. Proc Natl Acad Sci U S A.

[14] Eckfeldt CE, Mendenhall EM, Flynn CM, Wang TF, Pickart MA (2005). Functional analysis of human hematopoietic stem cell gene expression using zebrafish.. PLoS Biol.

[15] Pellagatti A, Cazzola M, Giagounidis AA, Malcovati L, Porta MG (2006). Gene expression profiles of CD34+ cells in myelodysplastic syndromes: involvement of interferon-stimulated genes and correlation to FAB subtype and karyotype.. Blood.

[16] Breit S, Nees M, Schaefer U, Pfoersich M, Hagemeier C (2004). Impact of pre-analytical handling on bone marrow mRNA gene expression.. Br J Haematol.

[17] Ge X, Yamamoto S, Tsutsumi S, Midorikawa Y, Ihara S (2005). Interpreting expression profiles of cancers by genome-wide survey of breadth of expression in normal tissues.. Genomics.

[18] Gutierrez NC, Lopez-Perez R, Hernandez JM, Isidro I, Gonzalez B (2005). Gene expression profile reveals deregulation of genes with relevant functions in the different subclasses of acute myeloid leukemia.. Leukemia.

[19] Cheok MH, Yang W, Pui CH, Downing JR, Cheng C (2003). Treatment-specific changes in gene expression discriminate in vivo drug response in human leukemia cells.. Nat Genet.

[20] Affymetrix annotation files, provided by manufacturer, accessed from www.affymetrix.com. Affymetrix, Inc.

[21] Ramalho-Santos M, Yoon S, Matsuzaki Y, Mulligan RC, Melton DA (2002). “Stemness”: transcriptional profiling of embryonic and adult stem cells.. Science.

[22] Dettling M, Buhlmann P (2002). Supervised clustering of genes.. Genome Biol.

[23] Tusher VG, Tibshirani R, Chu G (2001). Significance analysis of microarrays applied to the ionizing radiation response.. Proc Natl Acad Sci U S A.

[24] Heaney ML, Golde DW (1999). Myelodysplasia.. N Engl J Med.

[25] Tan PK, Downey TJ, Spitznagel EL, Xu P, Fu D (2003). Evaluation of gene expression measurements from commercial microarray platforms.. Nucleic Acids Res.

[26] Fortunel NO, Otu HH, Ng HH, Chen J, Mu X (2003). Comment on “ ‘Stemness’: transcriptional profiling of embryonic and adult stem cells” and “a stem cell molecular signature”.. Science.

[27] Marshall E (2004). Getting the noise out of gene arrays.. Science.

[28] Ein-Dor L, Zuk O, Domany E (2006). Thousands of samples are needed to generate a robust gene list for predicting outcome in cancer.. Proc Natl Acad Sci U S A.

[29] Dobbin KK, Beer DG, Meyerson M, Yeatman TJ, Gerald WL (2005). Interlaboratory comparability study of cancer gene expression analysis using oligonucleotide microarrays.. Clin Cancer Res.

[30] Irizarry RA, Warren D, Spencer F, Kim IF, Biswal S (2005). Multiple-laboratory comparison of microarray platforms.. Nat Methods.

[31] Larkin JE, Frank BC, Gavras H, Sultana R, Quackenbush J (2005). Independence and reproducibility across microarray platforms.. Nat Methods.

[32] Shi L, Reid LH, Jones WD, Shippy R, Warrington JA (2006). The MicroArray Quality Control (MAQC) project shows inter- and intraplatform reproducibility of gene expression measurements.. Nat Biotechnol.

[33] Choi JK, Yu U, Kim S, Yoo OJ (2003). Combining multiple microarray studies and modeling interstudy variation.. Bioinformatics.

[34] Rhodes DR, Yu J, Shanker K, Deshpande N, Varambally R (2004). Large-scale meta-analysis of cancer microarray data identifies common transcriptional profiles of neoplastic transformation and progression.. Proc Natl Acad Sci U S A.

[35] Ivanova NB, Dimos JT, Schaniel C, Hackney JA, Moore KA (2002). A stem cell molecular signature.. Science.

[36] Chen J, Archer TK (2005). Regulating SWI/SNF subunit levels via protein-protein interactions and proteasomal degradation: BAF155 and BAF170 limit expression of BAF57.. Mol Cell Biol.

[37] Bernstein BE, Mikkelsen TS, Xie X, Kamal M, Huebert DJ (2006). A bivalent chromatin structure marks key developmental genes in embryonic stem cells.. Cell.

[38] Mermel CH, McLemore ML, Liu F, Pereira S, Woloszynek J (2006). Src family kinases are important negative regulators of G-CSF-dependent granulopoiesis.. Blood.

[39] Karur VG, Lowell CA, Besmer P, Agosti V, Wojchowski DM (2006). Lyn kinase promotes erythroblast expansion and late-stage development.. Blood.

[40] Lindsey R, Momany M (2006). Septin localization across kingdoms: three themes with variations.. Curr Opin Microbiol.

[41] Chen Y, Liu W, McPhie DL, Hassinger L, Neve RL (2003). APP-BP1 mediates APP-induced apoptosis and DNA synthesis and is increased in Alzheimer's disease brain.. J Cell Biol.

[42] Ishida H, Masuhiro Y, Fukushima A, Argueta JG, Yamaguchi N (2005). Identification and characterization of novel isoforms of human DP-1: DP-1{alpha} regulates the transcriptional activity of E2F1 as well as cell cycle progression in a dominant-negative manner.. J Biol Chem.

