# Supplementary material for: Meta-Analysis of Microarray Studies Reveals a Novel Hematopoietic Progenitor Cell Signature and Demonstrates Feasibility of Inter-Platform Data Integration
Source: PLoS One. 2008 Aug 13;3(8):e2965. doi: 10.1371/journal.pone.0002965 (PMC2495035; doi:10.1371/journal.pone.0002965)
Supplement: Table S1 — (0.04 MB DOC) [file pone.0002965.s001.doc]

| **Supplementary Table 1: List of HSC genes that are highly expressed in 3 independent microarray experiments conducted using Nimblegen array platform.** | | |
| --- | --- | --- |
|  |  |  |
| **GENE** | **Mean GE (Log2)** | **Coefficient**  **of Variation** |
| caspase 8, apoptosis-related cysteine peptidase | 10.02068 | 0.048468 |
| thyroid hormone receptor associated protein 1 | 10.15983 | 0.255477 |
| lectin, galactoside-binding, soluble, 8 (galectin 8) | 11.5053 | 0.156001 |
| growth factor receptor-bound protein 10 | 10.73417 | 0.035451 |
| Chromosome 18 open reading frame 1 | 10.10393 | 0.16559 |
| eukaryotic translation initiation factor 5B | 10.11161 | 0.116548 |
| ubiquitin protein ligase E3A (human papilloma virus E6-associated protein, Angelman syndrome) | 14.53737 | 0.066485 |
| A kinase (PRKA) anchor protein 8 | 10.66927 | 0.056544 |
| ribonuclease H1 | 11.22847 | 0.105218 |
| CD55 molecule, decay accelerating factor for complement (Cromer blood group) | 10.39991 | 0.101494 |
| DNA segment on chromosome X and Y (unique) 155 expressed sequence, isoform 1 | 10.55549 | 0.085138 |
| ubiquitin specific peptidase 4 (proto-oncogene) | 10.54246 | 0.031547 |
| Rtf1, Paf1/RNA polymerase II complex component, homolog (S. cerevisiae) | 10.26807 | 0.218954 |
| SCC-112 protein | 11.1134 | 0.123723 |
| Transcription factor Dp-2 (E2F dimerization partner 2) | 10.0055 | 0.151676 |
| mannosidase, alpha, class 2A, member 2 | 10.28721 | 0.133046 |
| SWI/SNF related, matrix associated, actin dependent regulator of chromatin, subfamily e, member 1 | 10.03432 | 0.150485 |
| v-yes-1 Yamaguchi sarcoma viral related oncogene homolog - Lyn kinase | 10.69807 | 0.024775 |
